# An Active Approach to Behavior: Exergaming Bike Breaks for Students with Disabilities

**DOI:** 10.3390/bs16081344

**Published:** 2026-08-05

**Authors:** Sara Kupzyk, Heidi K. Reelfs, Kevin Kupzyk

**Affiliations:** 1 Department of Psychology, University of Nebraska at Omaha, Omaha, NE 68182, USA; 2 Department of Physical Therapy, Munroe-Meyer Institute, University of Nebraska Medical Center, Omaha, NE 68106, USA; heidi.reelfs@unmc.edu; 3 College of Nursing, University of Nebraska Medical Center, Omaha, NE 68198, USA

**Keywords:** physical activity breaks, exergaming, positive behavior intervention

## Abstract

Many children, particularly those with disabilities, do not meet the recommended amount of physical activity per day, which can increase the likelihood of poor health outcomes. Beyond the potential health benefits, physical activity breaks are associated with increased academic engagement and decreased stereotypy and off-task behavior. It is important to increase periods of physical activity in naturalistic settings like schools. The purpose of this article is to describe two pilot studies that examined the feasibility of integrating exergaming bike breaks into daily school routines for students with disabilities. Across the studies, data were collected on physical fitness, mood, classroom behavior, and acceptability. In Study 1, 13 students ages 9–13 with autism participated in exergaming biking for 15 min, 3 days per week for 6 weeks. No significant differences were observed between ride and non-ride days for teacher-reported behaviors; however, researcher-observed disruptive behaviors were significantly lower on ride days. In Study 2, six students with disabilities ages 9–17 who had a behavior intervention plan participated in biking for 5 days per week for 10 weeks. There was a significant increase in the percentage of time students were on-task and a decrease in time students were reported to be disruptive based on teacher behavior reports. Physical fitness remained low following intervention. Overall, exergaming biking was an acceptable and engaging form of physical activity that can be integrated into students’ daily routines. Use of the bikes was associated with some improvement in classroom behavior. We discuss implications for practice and recommendations for future research.

## 1. Introduction

It is recommended that children and adolescents engage in 60 min of moderate-to-vigorous physical activity daily (U.S. Centers for Disease Control and Prevention, 2024). However, only 20–28% of children and adolescents meet the recommended 60 min of physical activity, with the level of activity declining as children age (Physical Activity Alliance, 2024). Students with disabilities are less likely to meet the recommendations, with only 19% of children with developmental disabilities (DD) meeting the goal (Case et al., 2020; Ma et al., 2024). Limited physical activity of children with disabilities may be related to physical impairments (e.g., poor motor skills, balance, stability, gait and flexibility), psychological factors (e.g., social communication difficulties, sensory sensitivities, behavioral rigidity), and contextual variables (e.g., social environment, family and community, lack of access to resources) (Case et al., 2020; Borland et al., 2022; Rivera et al., 2025). There is a need to increase physical activity for individuals with disabilities using interventions that meet their needs. Increasing opportunities for acute physical activity may lead to improved health outcomes and have secondary benefits in the classroom. For example, researchers have identified a positive relationship between school-based physical activity and academic performance and improvements in executive functioning (Centers for Disease Control and Prevention, 2010; Rivera et al., 2025).

Schools are an important setting for encouraging physical activity for all students, including those with disabilities (Ha et al., 2025). Yet, students spend approximately 63% of their school day in sedentary activities (Egan et al., 2019). To address these concerns and promote healthier lifestyles, the Physical Activity Alliance (2024) recommended educating schools and families about the importance of physical activity, integrating physical activity into daily routines, tailoring interventions to meet the needs of demographic groups, and incorporating technology and media that promote physical activity. For programs to be feasible, it is important to embed physical activity into daily routines or programs, offer age-appropriate activities, and integrate choice (Bowling et al., 2021).

Students receiving special education services who have behavioral concerns may have a behavior intervention plan (BIP) as part of their individualized education program (IEP) that outlines positive, proactive approaches to address targeted concerns. Periodic breaks are a strategy that may be included in students’ plans to allow them time to reset and return to the classroom, thus preventing behavior concerns (Ennis & Lee, 2024). Although providing breaks can be effective, they often involve sedentary activities (e.g., sitting and watching an iPad, playing with Legos, talking with a teacher or peer, engaging in mindfulness practices). If breaks are planned into a student’s schedule, the breaks may be an ideal time for physical activity. In other words, physical activity can be a safe alternative for students with social, emotional, and behavioral concerns (Ash et al., 2017). Furthermore, antecedent exercise breaks are associated with decreased problem behavior, increased prosocial behaviors, and increased academic engagement (Greenspan et al., 2019).

Offering breaks that involve physical activity, however, can present logistical challenges in school settings. Interventions that involve walking or jogging can be impractical given space availability and scheduling conflicts that often arise in schools. For example, the gym and cafeteria are typically used by classes throughout the day; hallways can be busy or unsafe for moderate to vigorous forms of activity; and the availability of staff to supervise the activity outside of the classroom is limited. It is important to find ways to make acute physical activity breaks possible and appealing to staff and students in school settings.

Technology can be used to encourage engagement and reduce implementation burden on teachers or other school staff (Ha et al., 2025). Exergaming can be defined as “interactive video games combining physical activity with gaming” (Rosi et al., 2025, p. 1). Exergaming is associated with motor, cognitive, and psychological benefits (Rosi et al., 2025). In addition, exergaming appears to be a motivating activity for students with disabilities because of the engaging video game aspects that include rewards/points for movement and meeting targets (Stanish et al., 2017; Bowling et al., 2021). Exergaming biking is an interactive exercise that combines stationary biking with playing an integrated video game displayed on a connected monitor. Stationary bikes are likely to be a good fit for school settings for individual student breaks because they require minimal physical space.

Emerging research has indicated positive benefits of exergaming biking. For example, Bowling et al. (2016) implemented a seven-week exergaming biking intervention for children with behavioral health disorders as part of a regular physical education (PE) class at a therapeutic day school. Their results showed that the intervention was implemented as intended 90 percent of the time, and staff indicated that the intervention was acceptable (low level of burden). Students showed high engagement when biking, rarely refused to ride when given the opportunity, met target heart rate levels, and almost half reported they would like to also use the bikes during breaks from class.

In a related study (Bowling et al., 2017a), one group of students rode exergaming bikes two times per week for seven weeks, while another group of children participated in their regular PE class. Children in the intervention group were significantly less likely to display disruptive behaviors such as impulsivity and emotional lability, with effects being most pronounced on days the students biked. Furthermore, the results indicated that the duration of biking was a more significant predictor of improvements in self-regulation and fewer behavioral related time out of class than how intensely they biked (Bowling et al., 2017b). However, the studies by Bowling and colleagues are limited because they were implemented in a therapeutic school setting where more specialized staff are available to support implementation. Data are needed to learn more about the feasibility, effectiveness and acceptability of such interventions in public school settings.

In a recent review of literature on technology-infused physical activity interventions in schools, Ha et al. (2025) noted a recent shift from using technology for monitoring interventions to the use of technology as an agent of change. They describe the need for additional research to examine strategies to integrate technology-infused interventions into schools in a sustainable manner that encourages high fidelity of implementation and maintains positive outcomes. Overall, there is a significant need to increase opportunities for physical activity in school settings using strategies that are tailored to meet students’ interests and needs and can be incorporated into students’ regular routines. Exergaming biking is one strategy that shows promise, as students have shown high levels of engagement when riding, as well as related improvements in behavior.

Based on the promising findings of exergaming as a strategy to increase physical activity and improve behavior, our team sought to extend the research on the feasibility and effectiveness of an exergaming biking intervention to public school settings for students identified with autism spectrum disorder (ASD) and students with BIPs as part of their IEP. We provide an overview of two projects that evaluated exergaming interventions. Specifically, we sought to answer the following research questions:

1. To what extent does a school-based exergaming bike intervention improve physical fitness, mood, and classroom behavior for autistic students and students with BIPs?
2. Do teachers and students rate the exergaming biking intervention as acceptable?

Consistent with prior literature, we hypothesized that students would show improved classroom functioning, mood, and health outcomes. We also hypothesized that teachers and students would rate the intervention acceptable given the engaging nature of the bikes.

## 2. Exergaming Bike Breaks in Schools—Feasibility Studies

### 2.1. Study 1

The purpose of this pilot project was to develop an exergaming program in collaboration with local public schools for students with ASD at the elementary and middle school levels. We investigated the effects of exergaming breaks on physical fitness, mood, classroom behavior (academic engagement and stereotypy), and acceptability.

#### 2.1.1. Participants and Setting

The second author met with prospective teacher participants and parents/legal guardians of student participants for informed consent. Each component of the consent form (e.g., purpose, risks, benefits, right to withdraw) was reviewed, and the participants were given the opportunity to ask questions and confirm their understanding of the study and their rights as research participants. Student participants also provided assent to participate.

Thirteen students (11 males and 2 females; 12 White and 1 Asian American) from four schools (three elementary and one middle school; percent free/reduced lunch ranged from 30 to 92%), between the ages of 9 and 14, with a medical or educational verification of autism participated. The students all spoke English, were verbal, and were able to follow one-step directions. In addition, all students were able to physically sit on the stationary bike and pedal for at least one minute at a self-selected pace (i.e., had no significant motor impairment or activity restrictions that would require modifications or adaptations for stationary biking). They had no medical exemptions for physical education in the last six months.

Sixteen teachers participated in the project (1 male and 15 females; age range 34 to 56). Teachers completed pre- and post-measures and recorded daily behavior of the students during the same period each day.

#### 2.1.2. Materials

An Expresso HD™ virtual reality exergaming stationary bike with an elive subscription (manufactured by Interactive Fitness, Austin, TX, USA; purchased online) was placed in a room in each collaborating school (e.g., back of a special education room, sensory room, small individual room). There were three separate worlds for the rider to explore: Treasure World, Explorer World, and Dragon World. Within the worlds, there is a series of games. Each game includes components of three tactical skills: speed, power, and tactics (a balance of speed and power) to create a high intensity interval training workout. The rider can earn points and can achieve mastery of the game through many variable combinations of time and each tactical skill. For example, the rider can steer and pedal to move their pirate ship to capture the treasure before the flying dragons reach it. In other games, they might encounter large animals or a copious number of coins to gather. As the rider progresses through the training, they earn trophies and unlock new maps.

Other materials included floor mats, wireless headphones (listen to music while riding), a poster with step-by-step directions for exergaming biking, laminated perceived rate of exertion (PRE) and mood scales, and small prizes for riding (e.g., sticky hand, bouncy ball) as well as for completing the program (i.e., water bottle).

#### 2.1.3. Measures

**Progressive Aerobic Cardiovascular Endurance Run (PACER)**. Aerobic fitness was measured at pre- and post-intervention using the PACER test, which involves continuous running between lines 20 m apart in time to recorded beeps. The time between recorded beeps decreases at each level. Students continue until they are unable to keep pace with the beeps. There are 21 levels. A point is scored for each 20 m distance covered. This assessment was selected because it was familiar to the students, as it was used as a measure of physical fitness in their general physical education class.

**Target Heart Rate and Perceived Rate of Exertion Scale.** A heart-rate monitor was used to assess the child’s normal heart rate while lying quietly for 5 min. The target heart rate was calculated to be 60–80% of the age-predicted maximum heart rate. Students were prompted to hold the handles of the bike at 5, 10, and 15 min during the ride so that their heart rate could be recorded. A 10-point perceived rate of exertion visual tool shows students how hard they work during exercise. Students were prompted to indicate their perceived rate of exertion at 5, 10, and 15 min into the ride.

**Ride Information.** Data obtained from the Expresso HD^TM^ stationary bike included the date and time of day, course selected, duration of the ride, heart rate, power, cadence, and distance.

**Mood Rating.** Students reported their mood using the Zones of Regulation visual scale (Kuypers, 2026) before and after each ride. This method was selected because students were familiar with the scale as it was already used in the schools. Students selected between four color categories that corresponded to levels of control/regulation: blue—low/moving slowly, green—regulated/in control, yellow—heightened/starting to lose control, and red—extremely heightened/not in control.

**Classroom Behavior.** Teachers completed a daily behavior rating (Direct Behavior Rating [DBR]; Chafouleas & Riley-Tillman, n.d.) of the students’ behavior (i.e., academic engagement, stereotypy, and disruptive behavior) for 30 min following the designated biking period on ride and non-ride days. For each target behavior, the teacher assigned a percentage of time the student engaged in the behavior during the period (ranging from 0 to 100% of the time). Research assistants conducted periodic 20 min direct observations of classroom behavior using interval recording methods (Johnston & Pennypacker, 2009). Specifically, momentary time sampling using 10 s intervals was used to measure academic engagement. Partial interval recording was used to measure disruptive behavior and stereotypy.

**Physical Activity Enjoyment Scale (PACES).** The PACES is a 16-item questionnaire used to assess the extent to which an individual enjoys doing any given physical activity, regardless of whether the activity is done for exercise or sport. The scale has good internal consistency and item-total correlations (Kendzierski & DeCarlo, 1991; Moore et al., 2009). Students completed the measure at pre- and post-intervention.

**Acceptability.** The Intervention Rating Profile-15 (IRP-15) was completed by teachers. This is a one-factor questionnaire that assesses perceptions of the general acceptability of interventions. Teachers rated each statement on a 6-point Likert scale, ranging from 1 (strongly disagree) to 6 (strongly agree). Negatively worded items were reverse-coded and then mean item ratings were calculated by dividing the sum of the ratings by the total number of items. The internal consistency of the IRP-15 is reported to be 0.98 (Kratochwill et al., 1995; Martens et al., 1985).

The students completed the Children’s Intervention Rating Profile (CIRP), which is a 7-item, one-factor questionnaire that assesses children’s perceptions of the acceptability of interventions using a 5-point Likert scale, ranging from 1 (disagree) to 5 (agree). Negatively worded items were reverse-coded, and then mean item ratings were calculated by dividing the sum of the ratings by the total number of items answered. The reported internal consistency of the CIRP ranges from 0.79 to 0.89 (Turco & Elliott, 1986; Witt & Elliott, 1985).

#### 2.1.4. Intervention Procedures

Prior to starting the biking intervention, the students completed the PACER and PACES. Students then participated in exergaming biking for 15 min, 3 days per week, for 6 weeks. Each ride day, an implementer at the school (e.g., physical therapist, graduate research assistant) helped the student set up and begin exercise (i.e., sitting on the bike, logging in, selecting a game, setting the duration of the ride). The students rode at a self-selected pace and were permitted to end the ride at any time during the 15 min intervention period. Any exergaming biking sessions missed that were due to legitimate reasons (e.g., absent from school) were made up by adding additional sessions to the end of the protocol. Throughout the biking period, we collected classroom behavior data on days biked and not biked. Students also reported their mood before and after biking. After 6 weeks of intervention, the PACER and PACES were administered again in addition to the acceptability rating scales (i.e., IRP and CIRP).

#### 2.1.5. Data Analysis and Results

Descriptive statistics were calculated on all study variables. Due to the small sample size and non-normal distributions, non-parametric tests were used for analysis. Relationships among variables were assessed via Spearman’s correlations, and Wilcoxon signed rank tests were used to assess differences between ride-day and non-ride-day average scores on classroom observations (disruptive behavior, stereotypy, compliance, etc.). Differences in the Zones of Regulation scale were examined descriptively from pre-ride to post-ride by calculating the proportion of rides where the student reported their mood as anything other than green (in control/happy). Changes throughout the study of ride data (e.g., power, cadence, and distance) were assessed using hierarchical linear models (HLMs). Also known as multilevel modeling, HLMs account for the nesting of rides within students, the varying number of rides, and the varying intervals between rides. Through the use of maximum likelihood estimation, all available data are utilized (data are not deleted listwise). Ride data were assessed for normality before performing each model. IBM SPSS version 30 was used for all statistical analyses.

**Progressive Aerobic Cardiovascular Endurance Run (PACER).** Seven of 13 students increased the number of laps run from pre to post on the PACER, 3 completed fewer laps, and 3 completed the same number of laps.

**Target Heart Rate and Perceived Rate of Exertion Scale.** Students met their target heart rate during all rides. Correlation between PRE and heart rate during the last five minutes of the ride was low (0.21), indicating that the students were not accurate reporters of their physical exertion level.

**Ride Information.** Students rode for an average of 14:33 min (with a maximum ride time of 15 min). Power, cadence, and distance showed little change over the intervention period. The HLMs showed no significant improvements over time for these outcomes. Although average power output showed a slight but significant decline (*p* = 0.04), cadence and distance were consistent over the course of the study.

**Mood.** Student mood data were available from before and after rides for 213 rides. Of the 45 rides in which the student reported a mood other than green prior to the ride, 33.3% (15) of the mood ratings were changed to green after the ride. Of the 168 rides with a green mood prior to the ride, 89.3% remained green and 10.7% (18) were changed to red, blue, or yellow after the ride.

**Direct Classroom Observations.** Nonparametric Wilcoxon Signed Rank tests showed no significant differences between ride and non-ride days for teacher-reported academic engagement or stereotypy. Mean differences, however, were in the expected direction (i.e., higher engagement and lower stereotypy on ride days). Although no changes were observed in the teacher-reported disruptive behavior, researcher-observed disruptive behaviors were significantly higher on non-ride-days (Mean = 6.2) than ride days (Mean = 3.6) (*p* = 0.023).

**Physical Activity Enjoyment Scale (PACES).** There was no significant change in student reporting of physical activity enjoyment (pre 4.0 and post 3.95; highest score being 5). Eleven of the thirteen students (84.6%) reported that the bike was fun, one reported the bike to be hard, and one reported the bike to be boring. Forty-six percent of the students reported that they would like to use the bikes during breaks from class. After riding, 53.8% reported feeling more energy, 61.5% reported feeling more relaxed, and 53.8% reported thinking more clearly.

**Acceptability**. The mean teacher report on the Intervention Rating Profile was 3.8 (range from 1.7 to 5.9; scale range 1 to 6). Nine teachers indicated acceptability of the intervention, two reported neutral acceptability, and two reported moderate disagreement that the intervention was acceptable for the students (e.g., behavior concern not significant enough to warrant intervention). The students reported high levels of acceptability on the Children’s Intervention Rating Profile (average score was 4.6 out of 5).

#### 2.1.6. Discussion

This study provided initial evidence for an engaging form of physical activity for students with ASD that can be implemented in school settings where space is limited. The results indicated that the students met their target exercise heart rate, rode the bike for most of the time allowed (average of 14:33 min), and reported a high level of acceptability of the program. Teachers reported a moderate level of acceptability. There was no change in physical fitness as measured using the PACER; students performed below the expected minimal level of fitness on the PACER (i.e., 23–41 laps) before and after the intervention. This finding is consistent with literature indicating that students with disabilities show limited physical fitness. To achieve significant improvements, students will likely need more frequent opportunities for physical activity. Given that the students were poor reporters of the PRE, researchers should consider using direct measures of activity level (e.g., heart rate) to ensure that students reach, but do not significantly exceed, their target heart rate. Students reported little change in their mood after riding. Student disruptive behavior was significantly lower on ride days based on researcher observation. There were no significant differences between ride and non-ride days for academic engagement or stereotypy, but means were in the expected directions for researcher and teacher observations.

During this project, we identified a few areas for additional consideration. First, the exergaming bikes we selected were too large for some of the elementary-aged students, which limited the number of students able to efficiently and comfortably ride. Second, to encourage ongoing use without researcher support, it was essential to collaborate with staff and identify specific staff to help students transition from the classroom to the space where the bike was located. Although the bikes can be placed in small areas or within classrooms, a teacher or paraeducator must be present to help the student begin and end riding. Third, we found that it was valuable to offer a choice to ride and games to play so that the bike was a motivating and enjoyable experience. Fourth, more frequent sessions and longer length of intervention are likely needed to achieve positive health (e.g., improvements on the PACER) and classroom outcomes (e.g., engagement). Lastly, the time periods designated for biking were established based on available schedules for the students and researchers, which may not have been the ideal times for the students to ride (i.e., based on convenience rather than function). It may be beneficial to plan the use of exergaming to better meet individualized needs within a BIP. 

### 2.2. Study 2

In the second study, we evaluated the use of the bikes with students with behavioral goals on their IEP. We also included students in middle and high school as they are more likely to be able to independently transition from one room to another with limited supervision. In addition, we increased the dosage from three days per week for six weeks to five days per week for 10 weeks. We examined changes over time in teachers’ daily behavior reports of students’ on-task and disruptive behaviors.

#### 2.2.1. Participants and Setting

Students were recruited from one elementary school, one middle school, and one high school. To be included, the students had to be receiving special education services (e.g., Autism, Other Health Impairment (ADHD), Emotional Disturbance, Specific Learning Disability) and have behavior goals on their Individualized Education Program (IEP). Five male students and one female student participated. The students ranged in age from 9 years 11 months to 17 years 11 months (average age 13). Informed consent was obtained in the same manner described in Study 1.

#### 2.2.2. Materials

Materials included the Expresso HD™ virtual reality exergaming stationary bike, floor mats, wireless headphones, a poster with step-by-step directions for exergaming biking, and a laminated mood scale.

#### 2.2.3. Measures

Like the first study, data were collected on ride information, mood, classroom behavior (teacher report only due to personnel restrictions), and acceptability (student form modified to be agree/disagree). In place of the PACER, we collected data on fitness using the Two Minute Walk Test (2MWT) because it provides a functional measure of ability to access the school environment. In addition, it required fewer resources for administration (i.e., it does not require a gym and can be administered by school staff).

**Two Minute Walk Test (2MWT).** The 2MWT measures self-paced walking ability and functional capacity by measuring the distance walked in two minutes (Bohannon et al., 2018). Normative values for the 2MWT indicate adequate endurance and cardiovascular fitness for participation in daily activities and community ambulation based on age. Participants were instructed to walk continuously, covering as much ground as possible for 2 min. The distance walked was recorded.

#### 2.2.4. Intervention Procedures

Before starting the biking intervention, the students completed the 2MWT and PACES. Students then participated in 15 min of exergaming biking during times when they had a typical break scheduled. Biking was scheduled 5 days per week for 10 weeks. During the intervention, a research assistant or bike implementer at the school (e.g., physical therapist) met with the student daily for intervention. They helped the student set up and begin exercise (i.e., sit on the bike, log in, select a game, set the duration of the ride). The students rode at a self-selected pace and were permitted to end the ride at any time. Students rated their mood before and after each ride. Any exergaming biking sessions missed that were due to legitimate reasons (e.g., absent from school) were made up by adding additional sessions to the end of the protocol. After 10 weeks of intervention, the 2MWT, PACES, and acceptability rating scales were administered (i.e., IRP and CIRP).

#### 2.2.5. Data Analysis and Results

Similar to the first study, descriptive statistics were calculated on all study variables. Change over time was assessed using HLMs.

**Two Minute Walk Test (2MWT).** Five out of seven students increased the distance walked from pre- to post-testing on the 2MWT. The other two students decreased the distance walked from pre to post-testing on the 2MWT. All of the students fell below the average distance walked for their age groups on the pre- and post-2MWT.

**Ride Information.** Fifty-seven percent of student rides were over 10 min, with the average ride time being 10 min 9 s. Power, cadence, and distance showed minimal change over the intervention period.

**Mood.** When students initially rated their mood in the red or yellow zones (frustrated, angry), 65% reported an improvement to the blue or green zones (tired, happy) immediately after riding.

**Figure 1:**
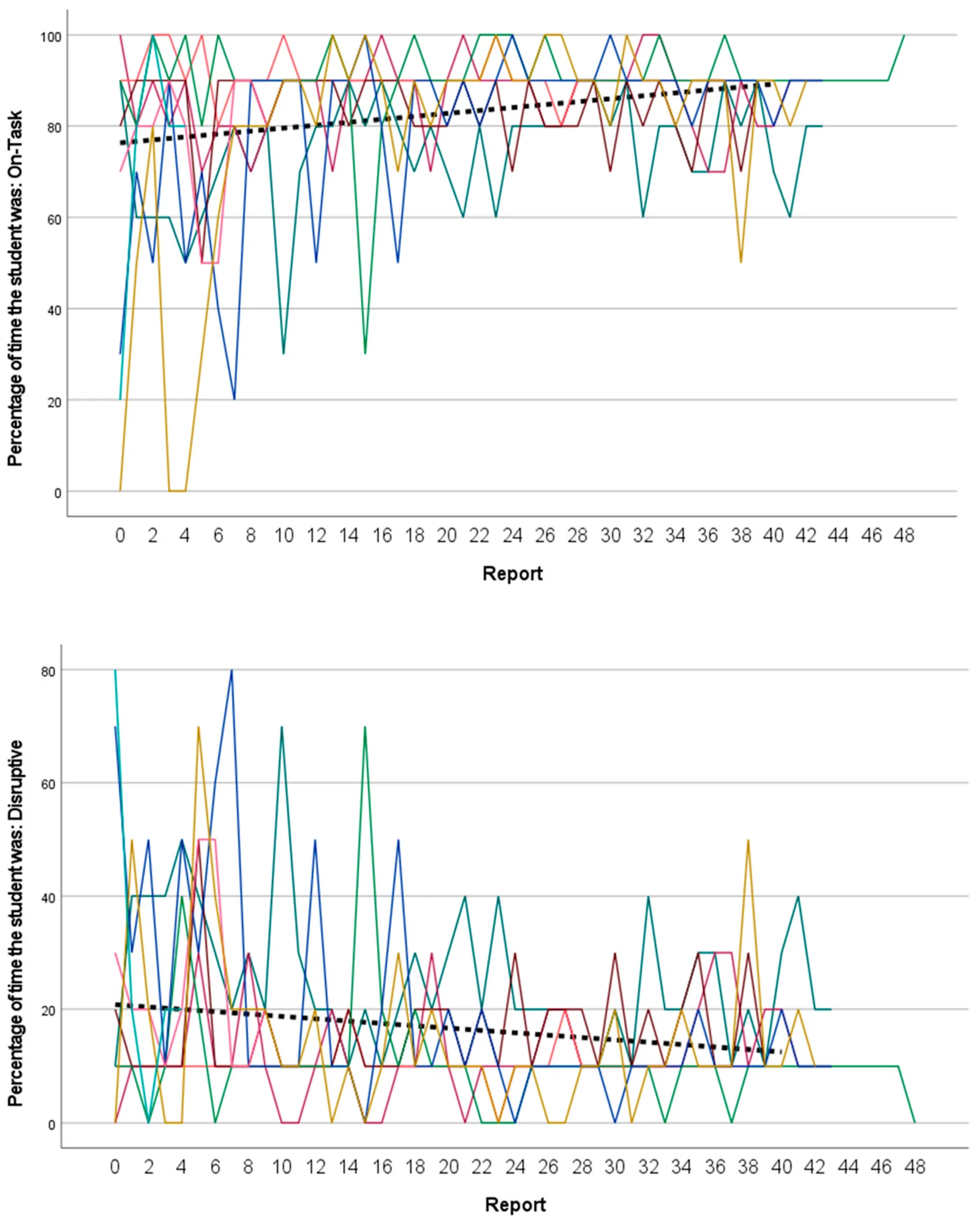
Study 2 Teacher Daily Behavior Report of the Percentage of Students’ On-Task and Disruptive Behavior. *Note.* The top panel shows teachers’ daily behavior reports of the percentage of on-task behavior by student over time (significant increase *p* < 0.001). The bottom panel shows teachers’ reports of the percentage of disruptive behavior (significant decrease *p* < 0.001). Each line represents a different student participant.

**Classroom Behavior.** Hierarchical linear models show a significant increase in the percentage of time students were on-task and a decrease in the time students were reported to be disruptive, based on teacher daily behavior reports (*p* < 0.001; Figure 1).

**Teacher and Student Acceptability.** The average score on the IRP was 4.25 out of 6 (1 = strongly disagree; 6 = strongly agree), indicating moderate acceptability. Four out of the six students reported liking biking and thought it would help others do better in school. Two of the students stated that they would continue biking if given the opportunity. Three of the students described biking as fun and one noted that they liked the music, games, and points. One student stated “At first I liked it, but then it took too long to unlock new games so it was frustrating”, and another stated they liked “nothing” about riding the bike.

#### 2.2.6. Discussion

As in Study 1, students were actively engaged during their riding time. They used most of the ride time available (i.e., an average of 10 min 9 s out of 15 min available). They also quickly learned how to log in and use the bike with little teacher/staff support. All students walked distances on the 2MWT that were below what was expected for their age. The physical endurance to cover more than 300 m is needed to walk independently in the community. Students with walking speeds below established norms on the 2MWT may lack the physical endurance to effectively access their school and community environments. This finding reiterates the need for additional opportunities for students with disabilities to improve their physical fitness. Generally, students reported positive mood before and after riding, with some increases reported. Teachers reported positive changes in classroom behaviors. Exergaming appears to be an acceptable intervention based on survey results. However, it is important to consider individual preferences and allow for choice so that the intervention is positive and exercise does not become aversive.

## 3. Conclusions

Most students do not meet the recommended level of physical activity per day (Physical Activity Alliance, 2024). Limited physical activity is even more pronounced for students with developmental delays (Case et al., 2020; Ma et al., 2024). Schools are an important setting for increasing students’ opportunities for physical activity. Beyond the potential for improved physical fitness, exergamining is associated with motor, cognitive, and psychological benefits (Rosi et al., 2025). Our findings indicate exergaming biking was an engaging form of physical activity (i.e., students rode for an average of 14 min in Study 1 and 10 in Study 2) that can be integrated into students’ daily routines. Furthermore, consistent with prior studies (Bowling et al., 2016; Bowling et al., 2017a, 2017b), use of the bikes was associated with some improvement in classroom behavior. Specifically, in Study 1, less disruptive behavior was observed by researchers on ride days and in Study 2, there was an increase in on-task behavior and decrease in disruptive behavior based on teacher reports. The interventions were generally acceptable to teachers and students. The format of exergaming also addresses some potential barriers for students with neurodevelopmental disorders. Specifically, the biking process provided a consistent routine, required little social communication, and offered options for students with sensory sensitivities (e.g., game selection, headphones, music). In addition, we were able to make small adjustments for the students with physical challenges to safely ride (e.g., foot straps). Students in these studies demonstrated poor physical fitness (PACER, 2-MWT), highlighting the need to incorporate additional opportunities for physical activity during the school day. Together, these studies provide initial evidence of the feasibility of acute physical activity breaks for students with ASD and behavioral concerns in school settings.

### Limitations and Future Directions

Given that these were pilot feasibility studies designed to provide preliminary evidence for the use of exergaming in schools, future research should address several limitations. First, these studies included a small sample of students with disabilities and did not include comparison groups. A larger sample of students with disabilities, with more diverse health and behavioral concerns, should be used to further examine outcomes of physical activity breaks. Future research should also include control and other physical activity comparison groups (e.g., running, exergaming videos, access to playground) to better control for threats to internal validity and understand components of the physical activity interventions that are most likely to lead to positive outcomes.

Second, the length of session (i.e., average of 10 and 14 min) and duration of the intervention (i.e., 6 and 10 weeks) was relatively brief. More frequent exergaming sessions and longer length of involvement may lead to more significant health outcomes. In addition, researchers may extend data collection to include additional health measures such as body mass index. Third, although teachers were given directions for completion of the daily behavior ratings, subjective ratings are at-risk for potential bias. Additional outcome measures such as work completion could be measured to further examine academic engagement.

Lastly, additional research is needed to better understand the mechanisms underlying the effectiveness and acceptability of acute exercise interventions. For example, Davison et al. (2016) proposed a model in which acute exercise may lead to changes in cognitive functions (i.e., executive function and mood), which may improve meta-cognition (i.e., self-regulation), and thereby academic achievement and classroom behavior. Furthermore, physical activity enjoyment (e.g., measured using PACES or related tools) may be an important variable to explore. If students enjoy the physical activity, they may be more likely to continue to engage in the activity; if students report low enjoyment, they may be at risk of withdrawing from opportunities for physical activity (Moore et al., 2009). Therefore, researchers and practitioners might assess students’ preference for types of activity when designing activity breaks. Similarly, future research should explore methods for training teachers and other school staff to integrate physical activity within daily lessons.

Regarding practical limitations, the time periods designated for biking were established based on available schedules for the students and researchers, which may not have been the ideal times for the students to ride. In future research, it would be beneficial to plan for use of the exergaming bikes to better meet individualized needs. For example, some students may benefit from the exercise as an antecedent intervention to facilitate improved behavior and transitions whereas other students might benefit from cycling as a consequence to appropriate behaviors (e.g., opportunity to use the exergaming bike as a reinforcer contingent on meeting behavioral expectations). In addition, although the bikes can be placed in small areas or within classrooms, a teacher or paraeducator must be present to help the student begin and end riding. School administration must show a commitment to increasing opportunities for physical activity to devote staff to such a program.

Altogether, we found that the exergaming bikes were most likely to be used when there was (1) a high level of staff and student interest and enthusiasm for using the exergaming bike, (2) the exergaming bike was easily accessible, (3) student schedules were somewhat flexible (i.e., easier when in a self-contained or resource classroom as opposed to students moving from room to room for classes unless breaks were explicitly part of their plan), and (4) exergaming biking was part of the students’ regular daily routine. To maintain a positive approach to behavior, we recommend giving students a choice of activities for their breaks. School teams interested in implementing acute physical activity during scheduled breaks should outline guidelines for use. A sample team planning form is included in the Supplementary Materials. The team should also consider funding mechanisms for purchasing and maintaining equipment and methods for ongoing evaluation. School teams will benefit from additional research that evaluates the long-term effects of school-based physical activity breaks for students with disabilities.

## Data Availability

Data are available upon request from the corresponding author.

## Supplementary Materials

The following supporting information can be downloaded at: https://www.mdpi.com/article/10.3390/bs16081344/s1, Table S1: Sample School Team Planning Form for Exergaming Bike Breaks.

## Abbreviations

The following abbreviations are used in this manuscript:

ASD	Autism Spectrum Disorder
DD	Developmental disabilities
IEP	Individualized education program
BIP	Behavior intervention plan
PE	Physical education
PRE	Perceived rate of exertion
