# Supplementary material for: An Active Approach to Behavior: Exergaming Bike Breaks for Students with Disabilities
Source: Behav Sci (Basel). 2026 Aug 5;16(8):1344. doi: 10.3390/bs16081344 (PMC13509195; doi:10.3390/bs16081344)
Supplement: Supplementary file 1 [file behavsci-16-01344-s001.zip › behavsci-4362630-supplementary.pdf]

Supplemental Material

Table S1. Sample School Team Planning Form for Exergaming Bike Breaks.

**Exergaming Bike Breaks**	
**Location of Exergaming Bike(s)**	
**Primary bike coordinator**	
**Student indicators for bike use**	☐ Size appropriate for the bike<br>☐ No medical concerns that prevent exercise/no medical exemption from physical education in the last six months or significant motor impairments<br>☐ Student is able to follow at least one-step directions<br>☐ During a practice ride, the student was able to move the pedals and steer<br>☐ Student reports interest in riding<br>☐ Student has a behavior intervention plan with breaks or team determines the student might benefit from biking and has flexibility in schedule for breaks
**How exergaming biking might be used/opportunities given to a student**	☐ Antecedent intervention to prevent a behavior<br>☐ Plan to use the bike before times when problem behavior is more likely each day<br>☐ Consequent intervention to reinforce appropriate target behavior

	☐ Plan to use the bike when the student meets expectations/goal for behavior each day<br>☐ Plan to use the bike when the student appropriately requests a break<br>☐ Noncontingent time-based<br>☐ Plan to use the bike at set times throughout the day regardless of behavior
**Permission considerations**	☐ Acute physical activity breaks will be written in the child's IEP or BIP<br>Or<br>☐ Parental permission for bike will be obtained
**Evaluation data to collect**	☐ Daily behavior report<br>☐ Individual student graphs of target behavior progress<br>☐ Student and teacher acceptability<br>☐ Use of bikes
**Daily Implementation Checklist**	☐ Supervise students during the use of the exergaming biking.<br>☐ Prompt or help the student log in with their individualized username and password.<br>☐ Student rides for designated length of time.<br>☐ Student returns to class.
